# An intra-droplet particle switch for droplet microfluidics using bulk acoustic waves

**DOI:** 10.1063/1.4984131

**Published:** 2017-05-26

**Authors:** Anna Fornell, Mathias Ohlin, Fabio Garofalo, Johan Nilsson, Maria Tenje

**Affiliations:** 1 Department Biomedical Engineering, Lund University, Lund, Sweden; 2 Department Engineering Sciences, Uppsala University, Uppsala, Sweden; 3 Science for Life Laboratory, Uppsala University, Uppsala, Sweden

## Abstract

To transfer cell- and bead-assays into droplet-based platforms typically requires the use of complex microfluidic circuits, which calls for methods to switch the direction of the encapsulated particles. We present a microfluidic chip where the combination of acoustic manipulation at two different harmonics and a trident-shaped droplet-splitter enables direction-switching of microbeads and yeast cells in droplet microfluidic circuits. At the first harmonic, the encapsulated particles exit the splitter in the center daughter droplets, while at the second harmonic, the particles exit in the side daughter droplets. This method holds promises for droplet-based assays where particle-positioning needs to be selectively controlled.

## INTRODUCTION

Compartmentalization of biological assays in small droplets allows for improved analytical analysis.^1–3^ The small volumes make droplet-based platforms particularly useful for assays where reaction reagents are expensive, the sample is scarce, or there is an interest to probe large numbers of single cells.^4–8^ The miniaturization of biological assays does however require the integration of several unit operators capable of encapsulating particles,^9^ adding reagents,^10^ and analyzing the droplet content.^11,12^ Although the microfluidic platform allows for these to be integrated with a compact foot-print, further advantages could be obtained by increasing the complexity of each circuit in analogy to the design of electronic or optical circuits. This calls for the need of microfluidic switches that can be included to direct the encapsulated particles into desired pathways. Consequently, methods to handle the content inside the droplets with high recovery are also needed.

In the previous work, acoustic,^13,14^ magnetic,^15–17^ and hydrodynamic forces^18–20^ have been used for intra-droplet particle manipulation. Of these, acoustic forces are particularly interesting, since acoustic methods allow for on-demand control and have the possibility to manipulate a variety of particles and cells. Acoustic forces have, since long, been reported to focus particles in one-phase systems,^21–24^ but it is first recently that acoustics have been applied for manipulation in two-phase systems.^25–27^ Previously, we reported on acoustic particle enrichment inside droplets using the first harmonic.^13^ However, using only the first harmonic may limit the applications of the technique since particles can only be aligned at the center-line of the droplets. In this paper, we present a device that combining an improved droplet-splitter and using the first and second harmonics enable direction-switching of encapsulated particles into either the center (following pathway 1) or the side daughter droplets (following pathway 2). This opens for on-demand direction of particles into different daughter droplets, further increasing the capability of droplet-based platforms.

## METHOD

**FIG. 1.**
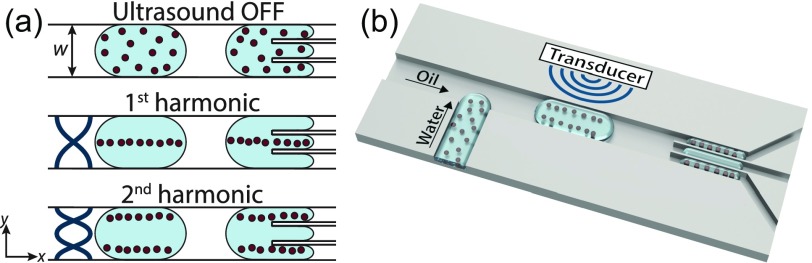
(a) Acoustic manipulation of particles inside droplets. (b) Acoustic-controlled positioning of particles (here shown for the second harmonic).

The operating principle is shown in Fig. 1. First, water-in-oil droplets containing particles are generated. Transducer activation generates vibrations in the device, and a standing wave is set between the channel walls at resonance. The standing wave induces the acoustic radiation force on the encapsulated particles that migrate towards the pressure nodal-lines.^28^ At the first harmonic, a single pressure nodal-line is generated at the center of the channel, and at the second harmonic, two pressure nodal-lines are generated at y=w/4 and y=3w/4, where w is the channel width [Fig. 1(a)]. By changing the frequency between the first and second harmonics, the particles can be directed to either the center or the side daughter droplets in a trident-shaped droplet-splitter.

The microfluidic channels were dry-etched in silicon and anodic bonded to a glass lid.^29^ The main channel is 370* × *100 *μ*m^2^. The droplet-splitter consists of two vertical side walls 10 *μ*m thick dividing the main channel into three equally large outlets. A piezoelectric transducer (0.7 mm thick, 2.9 MHz resonance frequency, Pz26, Ferroperm Piezoceramics) was glued on the chip and actuated by a function generator (33220A, Agilent Technologies) after amplification (75A250, Amplifier Research), and the voltage was 30 V_pp_. The dispersed phase was deionized water, and the continuous phase was olive oil. Polystyrene microbeads (10 *μ*m diameter, Sigma-Aldrich) or yeast cells (Kronjäst, Jästbolaget) were suspended in the aqueous phase. The flows were controlled by syringe pumps (NEMESYS, Cetoni). All experimental results were captured by a camera (XM10, Olympus) mounted on a microscope (BX51W1, Olympus). The system performance was evaluated by manually counting the beads from the videos.

## RESULTS AND DISCUSSION

**FIG. 2.**
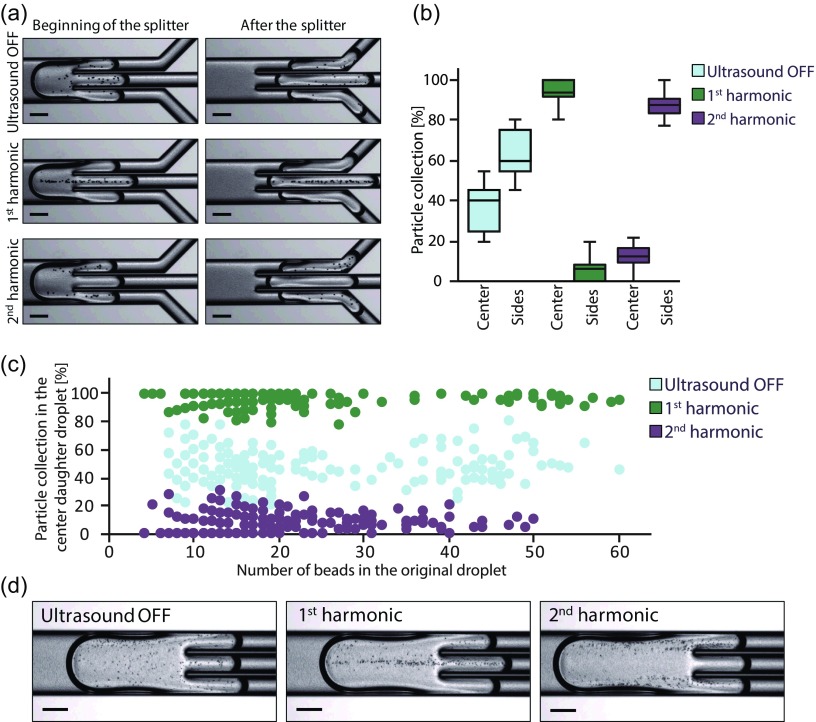
(a) Acoustic-controlled positioning of encapsulated polystyrene beads. Total flow rate is 9 *μ*l/min. (b) Box plot showing the system performance. The data are presented as the ratio of beads in the center or side daughter droplets compared with the total number of beads in the center and side daughter droplets. (c) Particle collection in the center daughter droplets compared with the total number of beads as a function of the bead concentration in the original droplet. Note that the “banding effect” visible at low bead counts occurs since the quantization step per bead is relatively large there. In both (b) and (c), N = 180 droplets in each experiment. (d) Acoustic-controlled positioning of yeast cells. Total flow rate is 3 *μ*l/min. All scale bars: 150 *μ*m.

A microfluidic chip for intra-droplet particle switching has been evaluated. In the first experiment, droplets containing polystyrene beads were generated, and each droplet was split into three daughter droplets. The fluid flows were adjusted to give daughter droplets of approximately the same volume (6 nl). Figure 2(a) shows the effect of applying ultrasound. At the first harmonic (1.83 MHz), the beads were moved towards the center-line of the droplets, and this resulted in the enrichment of particles in the center daughter droplets. To allow for particle switching, the actuation frequency was increased to the second harmonic (3.67 MHz) and the beads were moved into two pressure nodal-lines instead, causing the beads to be enriched in the side daughter droplets.

The number of beads in the daughter droplets was counted, and the results are presented in Figs. 2(b) and 2(c). Without ultrasound, the beads were distributed in all daughter droplets. It is however noted that the distribution is not completely equal, i.e., 1/3 in the center and 2/3 in the sides, and this might be attributed to small variation in volume between the center and side daughter droplets, and internal fluid advection in the droplets.^19,20,30^ However, at acoustic actuation, a pronounced difference between the center and side daughter droplets was observed. At the first harmonic, the system showed 96 ± 5% collection of beads in the center daughter droplets, whereas at the second harmonic, 92 ± 7% of the beads were collected in the side daughter droplets. The system showed high performance regardless of the initial bead concentration [Fig. 2(c)]. In the supplementary material, a video shows the direct effect of switching the frequency as the beads are immediately moved into opposite daughter droplets.

There are mainly three effects affecting the particle-positioning within the droplets: acoustic forces, internal fluid advection, and particle sedimentation. Sedimentation is mostly relevant in slow-moving droplets, while the internal advection increases with the flow rate.^30^ Consequently, there is an upper limit when the acoustic force is not strong enough to compete with the rapid mixing. The acoustic radiation force is proportional to the applied voltage squared,^28^ and depending on the set-up and the intended application, the voltage may need to be constrained to avoid excessive heating. The presented system has been evaluated with good acoustic focusing at 3–18 *μ*l/min (see supplementary material). In addition to direct effects on the acoustic particle-positioning, the total flow rate also affects the droplet length,^31^ which in turn influences the droplet splitting. This effect seems to have a more pronounced impact on the enrichment performance than the increased advection at higher flows.

The presented method can also be applied to direct yeast cells into different daughter droplets [Fig. 2(d)]. The fact that the method can handle cells without labelling makes the technology favorable for biological applications and is advantageous compared to magnetic manipulation.^15–17^ The proposed system shows better performance compared with hydrodynamic methods as these require large and heavy particles and/or very low flows to achieve acceptable particle accumulation.^18–20^ However, the most striking difference is that no other method as of today can switch particles on-demand into different daughter droplets in a continuous operation. These aspects make acoustic particle manipulation using the first and second harmonics an essential tool for the development of novel droplet-based assays.

## CONCLUSION

We have demonstrated an acoustic method to direct particles into different daughter droplets. Using the first harmonic, the encapsulated particles exit the droplet splitter in the center daughter droplets, while using the second harmonic, the particles exit in the side daughter droplets instead. We show that polymer microparticles as well as yeast cells can be manipulated. The presented method expands the droplet microfluidic tool-box since the acoustic manipulation is the only available method that continuously can switch particles on-demand into different daughter droplets.

## SUPPLEMENTARY MATERIAL

See supplementary material for the video showing intra-droplet particle-switching (S1), and photographs of acoustic-controlled positioning at different total flow rates (S2).
